# Deep Features from Pretrained Networks Do Not Outperform Hand-Crafted Features in Radiomics

**DOI:** 10.3390/diagnostics13203266

**Published:** 2023-10-20

**Authors:** Aydin Demircioğlu

**Affiliations:** Institute of Diagnostic and Interventional Radiology and Neuroradiology, University Hospital Essen, Hufelandstraße 55, 45147 Essen, Germany; aydin.demircioglu@uk-essen.de

**Keywords:** radiomics, benchmarking, pretraining, features, stability, reproducibility, deep learning, machine learning

## Abstract

In radiomics, utilizing features extracted from pretrained deep networks could result in models with a higher predictive performance than those relying on hand-crafted features. This study compared the predictive performance of models trained with either deep features, hand-crafted features, or a combination of these features in terms of the area under the receiver-operating characteristic curve (AUC) and other metrics. We trained models on ten radiological datasets using five feature selection methods and three classifiers. Our results indicate that models based on deep features did not show an improved AUC compared to those utilizing hand-crafted features (deep: AUC 0.775, hand-crafted: AUC 0.789; *p* = 0.28). Including morphological features alongside deep features led to overall improvements in prediction performance for all models (+0.02 gain in AUC; *p* < 0.001); however, the best model did not benefit from this (+0.003 gain in AUC; *p* = 0.57). Using all hand-crafted features in addition to the deep features resulted in a further overall improvement (+0.034 in AUC; *p* < 0.001), but only a minor improvement could be observed for the best model (deep: AUC 0.798, hand-crafted: AUC 0.789; *p* = 0.92). Furthermore, our results show that models based on deep features extracted from networks pretrained on medical data have no advantage in predictive performance over models relying on features extracted from networks pretrained on ImageNet data. Our study contributes a benchmarking analysis of models trained on hand-crafted and deep features from pretrained networks across multiple datasets. It also provides a comprehensive understanding of their applicability and limitations in radiomics. Our study shows, in conclusion, that models based on features extracted from pretrained deep networks do not outperform models trained on hand-crafted ones.

## 1. Introduction

Radiomics has emerged as a promising image analysis technique, providing insights into the characterization and quantification of radiological imaging, especially supporting diagnostic and prognostic tasks [1,2,3]. Essentially, radiomics involves the application of a classical machine learning pipeline to process radiological data [4,5]. A central step is generating features from radiological imaging, since these are designed to capture the content in a comprehensive fashion. For this task, hand-crafted features based on morphology, intensity, and texture have been developed, stemming back from ideas in the field of image analysis in the 1970s and 80s [6,7]. Even though these features have proven successful, they might be suboptimal, since they were designed mainly for other domains. Thus, they might not fully capture information in radiological imaging.

Recently, deep learning models have been applied successfully in many areas [8,9,10]. A key advantage is that they can automatically learn relevant features from imaging data, which may be especially beneficial compared to hand-crafted features, leading to models with improved prediction and increased utility. Yet, a fundamental problem in applying these methods lies in the small sample sizes typically involved in radiomic datasets. Deep learning methods usually possess a large number of trainable parameters that are indispensable in learning features independently; however, at the same time, a large number of data are needed to train these parameters effectively. If the sample sizes are small, as in radiomics, they might not perform well.

A simple alternative is to use pretrained deep networks as feature extractors. In this approach, networks are first trained on other large datasets, such as everyday images or medical images that are not directly related to the existing data. Ideally, because of the larger amount of data, the network will learn features that represent the data more abstractly at a higher level. The hope is that these features are more general in nature and will work well on other datasets, too. In radiomics, these features could thus form a viable alternative to manually generated features and, in the best case, lead to better models.

However, a large number of pretrained models exist. These differ mainly in the dataset and the methods used during training. For example, many models are trained on the ImageNet dataset containing around 14 million images [11], but recently, networks pretrained on medical data were also introduced. In addition, training can be conducted in a supervised fashion and via self-supervision, which has the benefit of being an unsupervised method, e.g., no labels are needed. It is unclear which pretrained models should be used as feature extractors in radiomics, and whether they can provide an advantage over hand-crafted features. Similarly, the question is whether the two feature classes could complement each other, leading to even higher predictive models.

This study, therefore, utilized multiple pretrained deep neural networks and tested their predictive performance across multiple radiomic datasets. Our study aimed to answer which pretrained networks work better, and whether models fused using deep and hand-crafted features together can further improve performance. The major contribution of this research is conducting a comparative analysis of models trained on hand-crafted, deep features extracted from pretrained deep networks or a combination of these. A novelty is the utilization of a broad set of deep models comprising models trained on ImageNet (like DeiT III and Convnext-v2) and medical data (like MedicalNet). While the main metric is the area under the receiver-operating characteristic curve (AUC), we also employed other metrics for this comparison (like the Matthew correlation coefficient and F-score). This study is one of the first to compare these models over several datasets, allowing for a more robust assessment of their performance compared to studies using only a single dataset. It contributes to a more comprehensive understanding of the applicability and limitations of these approaches, and can guide future research and applications.

The remaining paper is organized as follows: Section 2 provides an overview of related works, while Section 3 describes the materials and methods used in this study. Section 4 presents the analysis results, and Section 5 discusses the results, providing concluding remarks and directions for possible future work.

## 2. Related Works

Studies comparing models using hand-crafted features and deep features have been conducted previously (Table 1), but nearly all these studies only compared them on single datasets. Since such comparisons cannot be readily generalized to other datasets, the results in the literature are accordingly mixed. While some studies have reported a decreased or no improvement at all (e.g., [12,13,14,15]), others reported improved performance when using deep features (e.g., [16,17,18,19,20]). The reasons for these differences are manifold and hard to identify. One reason could be the different validation schemes utilized. For example, Fu et al. employed an (uncommon) 4-fold CV [21], while Yan et al. used an internal cohort that was acquired at the same site [12]. In contrast, Feng et al. used an external cohort for their validation [19]. Yet, no clear pattern can be seen; for example, Feng et al. observed a gain of +0.10 in AUC, while Hou et al. also used an external cohort, but did not see any gain in using deep features [15]. Furthermore, even though many hand-crafted features were defined by the image biomarker standardization initiative [22] and thus should be comparable across different studies, some libraries for extracting hand-crafted features do not adhere to this standard, and can lead to differences in predictive performance between the studies. The same is true for the preprocessing steps; these can have a large impact on the performance, but there is no standardization. For example, normalization of the features using different methods like z-Score or min–max can influence the resulting model [23], but they differ between the studies.

A study comparing the predictive performance of multiple pretrained deep networks across multiple radiomics datasets still needs to be conducted.

## 3. Materials and Methods

For this retrospective study, several publicly available datasets were used; corresponding approvals from the ethical review boards have been granted. Ethical approval for this study was waived by the local ethics committee (Ethik-Kommission, Medizinische Fakultät der Universität Duisburg-Essen, Essen, Germany). All methods and procedures were performed following the relevant guidelines and regulations, the CheckList for EvaluAtion of Radiomics research (CLEAR) [27], and in accordance with the Declaration of Helsinki.

### 3.1. Study Design

**Figure 1:**
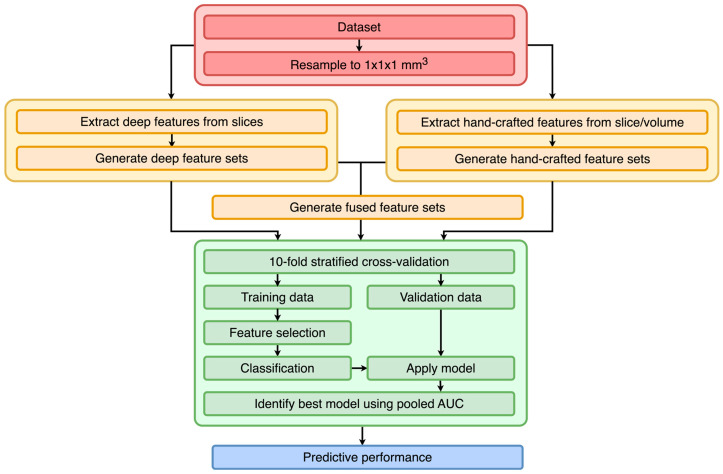
Overall flowchart of the study. For each dataset and pretrained neural network, a 10-fold stratified cross-validation is employed.

The study follows the standard radiomics pipeline very closely [3,28] and consists of preprocessing, feature extraction, selection, and classification (Figure 1). Briefly, the datasets were first preprocessed via resampling to a homogenous spacing. The volumes were then used to extract hand-crafted and deep features. The hand-crafted features were extracted directly from the volumes. For the deep features, first, slices were extracted. The slices were then processed using pretrained deep models, and the features were extracted from a layer of the networks. These steps resulted in multiple feature sets, which were then used for model building, consisting mainly of a feature selection and classification step. A 10-fold stratified cross-validation was utilized to determine which methods performed best. During cross-validation, the training data were normalized, and a feature selection algorithm was applied. On the resulting features, a classifier was trained. This classifier was then used to obtain predictions on the validation folds. These predictions were then pooled across the folds, and several metrics were computed.

### 3.2. Datasets

**Figure 2:**
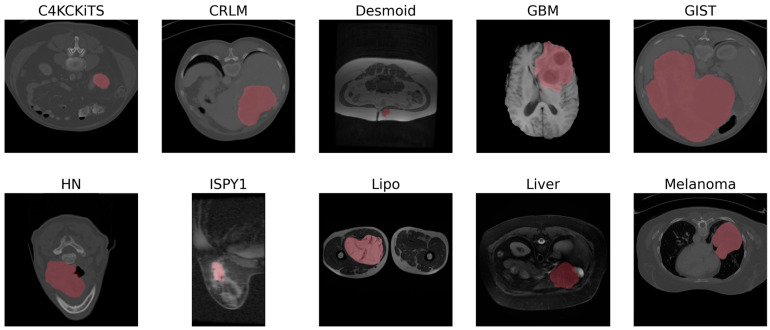
Example slices for each dataset. Images were normalized to the range between 0 and 255, regardless of whether they were CT or MR sequences. The segmentation is depicted in red. The HN image was cropped for visualization purposes.

Ten radiomic datasets were collected from the Workflow for Optimal Radiomics Classification (WORC) [29] and the cancer imaging archive (TCIA) [30] (Table 2). All datasets were binary, i.e., the outcome is either 0 or 1. Each dataset consisted of a scan (e.g., a CT or an MR sequence) and a segmentation of the volume of interest (VOI) (Figure 2). All samples were included, except for a few wherein either the slice thickness was comparatively too large (i.e., larger than twice the median slice thickness) or a technical error occurred while computing the hand-crafted feature vectors. All scans were resampled using bicubic interpolation to a homogenous spacing of 1 × 1 × 1 mm^3^. The segmentations were resampled to the same spacing, but using the nearest neighbor method to avoid partial volume effects.

### 3.3. Preprocessing

For each dataset, slices were extracted from the bounding box around the segmentation. Slices in which the segmentation occupied less than 50 pixels (around 7 mm^2^) were excluded, since the region-of-interest (ROI) would be too small to extract meaningful features. The same intensity normalization was applied per slice, regardless of whether a CT or MR dataset was processed. For the networks trained on ImageNet data, slices were normalized with respect to the mean and standard deviation of the ImageNet dataset. Slices for the RadImageNet were normalized by linearly scaling the intensities into the range [−1, 1]. For MedicalNet, the intensities of the volumes were z-Score normalized. In addition, the volume of interest was resized to 112 × 112 × 56. Slices were not further rescaled during preprocessing.

For the hand-crafted features and 3-D networks, the scan was cropped using a bounding box around the segmentation.

### 3.4. Feature Extraction

After preprocessing, hand-crafted and deep features were extracted from either the slices or the scans.

For the hand-crafted features, 2-D and 3-D features were extracted from the scans using PyRadiomics v3.1 [35]. For this, each scan was first processed via multiple preprocessing filters in addition to the unprocessed scans: Laplacian-of-Gaussian (with sigma 1.0. 2.0. 3.0. 4.0. 5.0), wavelet, square root, logarithm, square, exponential and gradient filter. Three types of features were extracted: morphological, intensity, and texture features. All feature classes were extracted that were available in PyRadiomics, namely, shape, firstorder, glcm (with the exception of SumAverage), glrlm, glszm, gldm and ngtdm. The computation of the features differed for CT and MRI. For MRI data, the intensities were normalized and scaled by 100. For CT data, no normalization was applied. For extraction, in both cases, the image values were quantized using a bin width of 25. Altogether, 1470 hand-crafted features were computed for each scan.

In addition to 3-D features, 2-D hand-crafted features were computed using the force2D option in PyRadiomics. This option ensured that all texture and intensity features were computed slice by slice. Altogether, five feature sets comprising hand-crafted features were computed. First, all features were extracted from the 3-D data (called “Hand-crafted, 3-D, All”). Therefore, these features can be regarded as the standard, and are used as the baseline for all comparisons. In addition, since morphological features are known to be important, a feature set comprising only morphological features was also generated (“Hand-crafted, 3-D, Morph”). Finally, a third feature set was created with all features except morphological ones (“Hand-crafted, 3-D, No morph”). In addition, two 2-D feature sets were generated: One comprising all 2-D features with the 3-D morphological features (“Hand-crafted, 2-D, Morph”), and one without (“Hand-crafted, 2-D, No morph”).

Extraction of the deep features from the pretrained neural networks was performed as follows [36]: Slices were first resized to 224 × 224 pixels and converted to 3-channel RGB images by adding the segmentation and a linear combination of the scan and segmentation as extra channels. Features were then extracted from the last convolutional layer using a global average pooling if necessary. In order to obtain a single feature vector for each patient, the features from all slices of a patient were merged using max pooling.

Several differently pretrained networks, mainly from the mmPretrain repository (https://github.com/open-mmlab/mmpretrain accessed on 26 August 2023), were used for the generation of deep features. First, four commonly used networks, trained on ImageNet-1K in a supervised manner, were employed: ResNet-34 [37], DenseNet-161 [38], VGG-16 [39] and Inception-V3 [40]. Second, networks pretrained on medical data were employed: The RadImageNet [41], which is a 2-D network pretrained on around 1.35 million CT, MRI, and ultrasound slices, and the MedicalNet [42], which is a 3-D network trained on 23 CT and MRI datasets. Both networks were trained with four different backbones (e.g., ResNet-10, Inception-V3). Third, the four best-performing network architectures from mmPretrained model zoo (https://mmpretrain.readthedocs.io/en/latest/modelzoo_statistics.html accessed on 26 August 2023) in terms of their top-5 accuracy were identified: Convnext-v2 [43], DeiT III [44], EfficientNet-b7 [45], and EfficientNetV2 [46]. All these involved pretraining in some form, e.g., in a non-standard or self-supervised fashion. Finally, four networks pretrained in a purely self-supervised way, i.e., without an additional finetuning step, were included: SimCLR [47], SimSiam [48], MoCoV3 [49], and Barlow twins [50]. These networks used the ResNet-50 as the backbone. A full list can be found in the Supplementary material, and more information about the networks can be found in the mmPretrain repository.

For each network, the corresponding features were extracted by max-pooling all slices. Additional feature sets were generated by combining the features with either morphological features only (“+Morph”) or with all hand-crafted features (“+All”). Since 20 pretrained networks were selected, this resulted in 60 feature sets involving deep and combined features.

### 3.5. Training

Each feature set was processed by a standard radiomics pipeline based on machine learning. First, the data were split into ten folds, ensuring the splits were the same across all feature sets to avoid any kind of bias from different subsamples. Then, for the training folds, the data was normalized using z-Scores. A feature selection was applied to remove redundant and irrelevant features. Five different methods were employed for this task [51]: ANOVA, Bhattacharyya, LASSO (with regularization parameter C = 1), random forest (with 100 trees), and t-Score. Because these methods do not select but only score the relevance of each feature, varying numbers of highest-scoring features were selected, ranging from 1, 2, 4, …, 64, 128. On the resulting feature set, one of three different classifiers was applied: logistic regression (with C chosen among 2^−8^, 2^−7^, …, 2^7^, 2^8^), neural networks (with three layers of size chosen among 16, 64, and 256), and random forests (with either 125, 250, or 500 trees).

### 3.6. Performance Metrics

Multiple metrics were used for comparison of the models. The primary metric used was the area under the receiver-operating characteristic curve (AUC), since this metric is rather insensitive to class imbalances in the data, and can be regarded as the de facto standard metric and is used in many radiomic studies. In addition, other metrics that are of interest to judge the quality of the model were employed: Accuracy, which measures the proportion of correctly predicted instances; recall, which quantifies a model’s ability to correctly identify positive instances; and precision, which calculates the proportion of true positive predictions among all positive predictions. In addition, the F-score, which is a harmonic mean of recall and precision, was measured [52]. Finally, we computed the Matthew correlation coefficient (MCC), which can be understood as a measurement that produces high values only if the model predicts both classes well [53].

### 3.7. Evaluation

For each model, its predictions on the left-out validation fold were computed. The model’s performance was then evaluated by pooling all predictions and computing the area under the curve of the receiving operator characteristic (AUC). The best-performing model for each feature set was then selected as the final model. These models were then studied with regard to the following two aspects: first, the performance gain of models using deep features compared to those using hand-crafted features; second, the improvement in performance resulting from adding morphological or all hand-crafted features to deep features. The hand-crafted model, which uses all features extracted from the 3D volume, was taken as a reference.

Statistical significance between the results was computed using a Wilcoxon signed-rank test. A *p*-value below 0.05 was considered to be statistically significant.

### 3.8. Experimental Settings

For all experiments and statistics, Python 3.8.10 was used. For statistical analysis, numpy (v1.24.4), scipy (v1.9.1), scikit-learn (v1.1.2), and scikit-posthocs (v0.7.0) were employed. For hand-crafted features, the Pyradiomics package (v3.10) was employed [35]. Deep models pretrained on ImageNet data were used from the mmPretrain v1.0.1 package; the RadImageNet and MedicalNet models, which are pretrained on medical data, were downloaded from their GitHub repository. PyTorch v2.0.1 and tensorflow v2.8.0 were used for the extraction of deep features. The code and data for all experiments can be found at https://github.com/aydindemircioglu/radSSL accessed on 13 September 2023. All experiments were performed on a workstation running Ubuntu 22.04.2 LTS and equipped with an AMD Threadripper 2950X with 128 GB of RAM and a Nvidia TITAN RTX graphics card with 24 GB of VRAM.

## 4. Results

**Figure 3:**
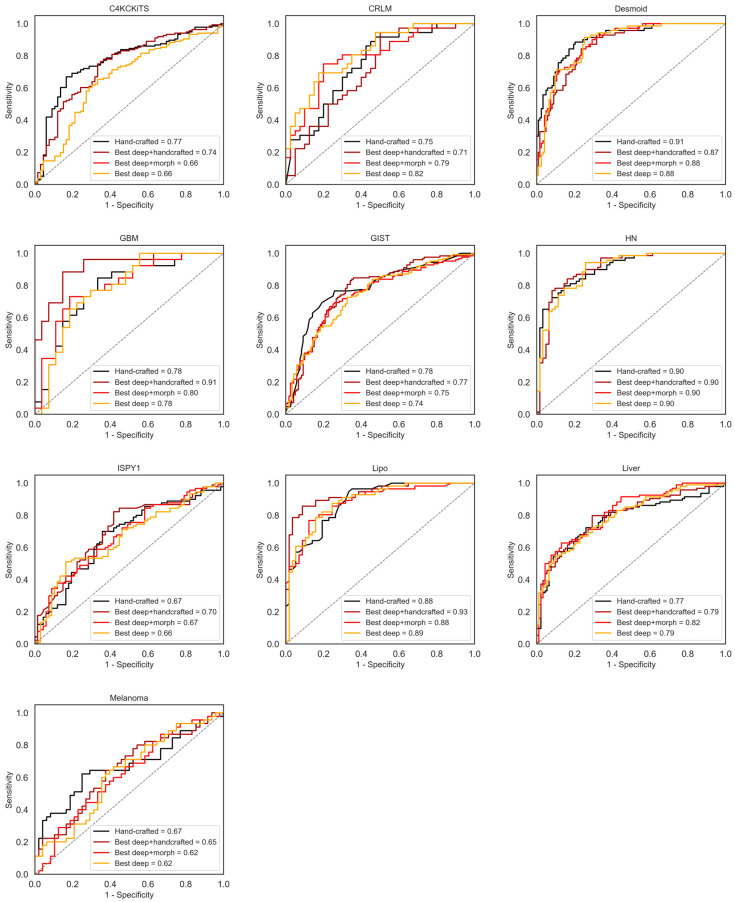
AUC curves for the standard model, “Hand-crafted, 3-D, All” (in black), the overall best-performing model using deep features (ConvNeXt V2, in yellow), using deep and morphological features (ConvNeXt V2, large, in red), and deep and hand-crafted features (SimSiam, ResNet-50, in magenta).

Overall, 65 feature sets were computed and analyzed. A graphical display of the AUC curves of the best models can be seen in Figure 3.

### 4.1. Hand-Crafted Features

Models that used all hand-crafted features with or without morphological ones performed very similarly overall, with the exception of the model based on morphological features alone (Table 3). The two models with 2-D features performed slightly better than those with 3-D features. The model that only employed morphological features performed worse, especially on the C4KC-KiTS dataset. However, it performed well on I-SPY1 and Lipo.

### 4.2. Deep Features

**Figure 4:**
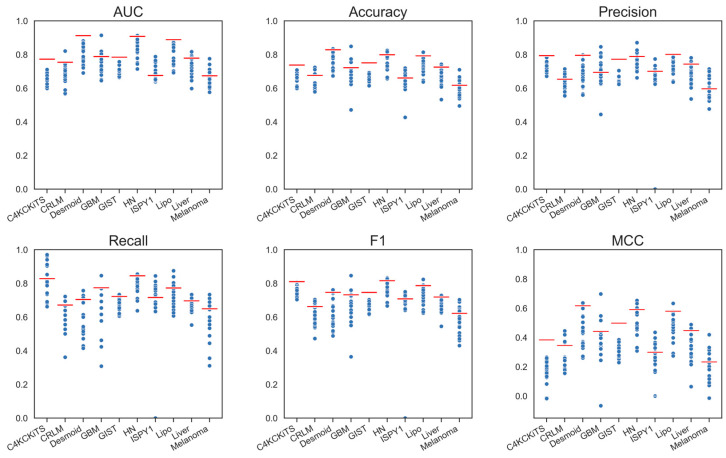
Graphical display of the performance metrics for all models using deep features. The red bars depict the performance of the standard model, “Hand-crafted, 3-D, All”.

Models using deep features performed slightly worse than those using standard hand-crafted features regarding AUC. Only the ConvNeXt V2 model achieved essentially the same performance as the hand-crafted radiomic model (Table 3, and Table S3 in the Supplementary Materials). Other models performed worse, and the loss in AUC was larger than 0.022. However, a larger difference could be seen in MCC for all models (Figure 4). These observations were also true for the networks pretrained on medical data (MedicalNet, RadImageNet), which performed inferior to the standard hand-crafted model (loss in AUC greater than 0.05).

### 4.3. Deep Features Fused with Morphological Features

**Figure 5:**
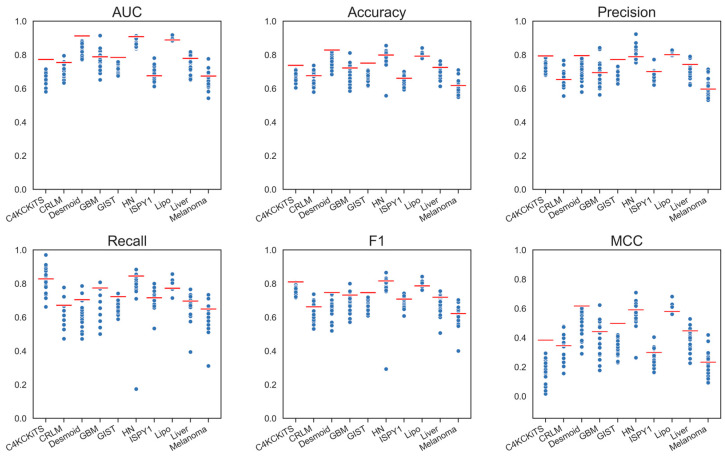
Graphical display of the performance metrics for all models using deep and morphological features. The red bars depict the performance of the standard model, “Hand-crafted, 3-D, All”.

Models using morphological features in addition to the deep features performed slightly better; the difference in AUC was statistically significant (+0.02 in AUC; *p* < 0.001) (Table 3 and Table S4 in the Supplementary Materials). Other metrics were also slightly better, although again, a performance drop in MCC could still be clearly seen (Figure 5). The best-performing model was still ConvNeXt V2, which virtually showed no improvement due to the morphological features, closely followed by SimSiam with ResNet-50 backbone, which gained 0.011 in AUC.

### 4.4. Deep Models Fused with All Hand-Crafted Features

**Figure 6:**
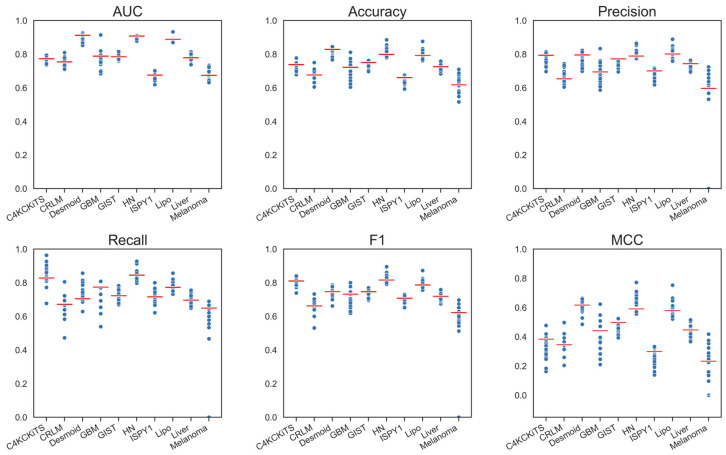
Graphical display of the performance metrics for all models using deep and all hand-crafted features. The red bars depict the performance of the standard model, “Hand-crafted, 3-D, All”.

Adding all hand-crafted features to the deep features improved the predictive performance further (Table 3 and Table S5 in the Supplementary Materials), and an overall difference of 0.034 in AUC over the models using only morphological features could be seen (*p* < 0.001). Compared to using only deep features, adding the hand-crafted features led to a higher improvement of 0.054 in AUC (*p* < 0.001). Yet, the previously best-performing model, ConvNeXt V2, showed only small improvements (+0.009 in AUC). In contrast, SimSiam showed a gain of +0.021 and performed slightly better than the standard, hand-crafted 3-D model (with a gain of +0.009). Regarding the other performance metrics, there was virtually no difference; this was also true for the MCC (Figure 6). While for several models, the gains in AUC compared to the standard hand-crafted model were minor but positive, the majority of the models still did not reach its level of performance.

## 5. Discussion

In this study, we investigated whether models using features extracted from pretrained deep neural networks can outperform models based on hand-crafted features. We utilized a large set of pretrained deep networks and compared them to ten radiomic datasets.

Our results indicate that, on average, the models using deep features do not improve over those using standard hand-crafted features. The overall best deep feature set, ConvNeXt V2, performed on average virtually the same; however, many other feature sets performed worse. It was also true for the features extracted from networks pretrained on radiological data and networks trained with better techniques (like self-supervised training), which can produce state-of-the-art results on ImageNet.

Adding morphological features did lead to a slight increase in overall performance, yet the best-performing model, the ConvNeXt V2, did not benefit. This observation starkly contrasts the intuition that morphological features, such as volume or sphericity, can improve the performance of 2-D networks, since these do not directly see the global shape. There could be two reasons why no such increase was observed: The networks can extract some morphological information about the lesions, even though only slices were processed. This might happen if the network extracts the segmentation size on each slice as a feature. On the other hand, it is also possible that for the datasets used in this study, the morphological information is not critical for the prediction, even though a recent study showed that volume is important in head and neck tumors [54].

Fusing all hand-crafted features with the deep features did lead to a slight performance boost. Even though this is reasonable, there are two reasons why this is somewhat surprising. First, adding all hand-crafted features will have doubled the number of features in many cases. Given the small sample size and the curse of dimensionality [55], more features could easily have led to diminished performance. Second, one could have expected that deep and hand-crafted features are complementary in nature. Thus, fusing both should have led to much higher-performing models. However, this was not observed. A reason for this could be that even though hand-crafted features could be sub-optimal, they have been shown in many cases to be highly discriminative. Deep features from pretrained networks might not be able to improve much over that. In addition, many outcomes might depend on information simply unavailable in the radiological data. Thus, regardless of whether hand-crafted or deep features are used, it cannot be expected that higher performance can be obtained.

Our results may also be interpreted in light of the ‘no free lunch’-theorem [56], which mainly states that no single classifier consistently outperforms other classifiers. Indeed, our results indicate that deep features can yield a higher gain over hand-crafted features for specific datasets. In other words, to obtain the highest predictive performance, the features must be further tuned to the dataset by some kind of training. Yet, there are two problems to consider: First, training involves many hyperparameters (e.g., the learning rate and schedule, the head architecture, augmentations, and regularization). Tuning them for a small dataset is a problem of its own, and can easily lead to overfitting when not handled properly. Second, a key advantage of using pretrained networks only as feature extractors is that the extracted features will be more reproducible [57]. In contrast, any training of a deep network, including finetuning, will inevitably destroy this property since the features will depend strongly on the dataset. In other words, training will decrease reproducibility substantially. Nonetheless, if predictive performance is the goal, it seems that using features extracted from pretrained deep networks is not very helpful, and some training should be conducted.

Although some studies exist that compare hand-crafted and deep features, these studies cannot be readily compared; therefore, we conducted a comparison analysis, where we can ensure comparability, e.g., that the preprocessing steps and cross-validation are the same for all feature sets. Our results also reflect the differences seen in the literature: If we consider a specific dataset and model (as is the case in nearly all of the above-cited studies), we can see either a large or nearly no difference between the models using hand-crafted and deep features. For example, on I-SPY1, the model using hand-crafted features obtained an AUC of 0.671, while the DeiT III model using all features obtained one of 0.788, indicating that the deep model is clearly better. Yet, had we used the ConvNeXt V2 model, we would have seen no difference in AUC (0.661 vs. 0.671). Therefore, we believe that a comparison across many datasets is key to understanding the performance of the models using pretrained deep features. However, as shown, we believe that for specific datasets and with further optimizations, the AUC of the deep models could be improved more [36]. Therefore, our observation underlines that deep features are not the panacea, but may be helpful for certain datasets [58].

Our study has certain limitations. First, we only used the pretrained deep networks as feature extractors. As discussed, finetuning them could lead to higher performance, albeit for a price. Second, nearly all networks we considered were two-dimensional. Such networks cannot directly utilize the spatial coherence present in the data. Simply adding morphological features did not help in this regard. Third, we only considered a single deep-learning pipeline. Tuning the extraction parameters for each dataset could increase the overall performance [36]. Furthermore, we used a simple cross-validation scheme, which might be prone to overfitting. However, all feature sets would be affected similarly, and it is reasonable to expect that the relative performances would more or less stay the same. In addition, it was shown that the amount of overfit for the classifiers we employed can be expected to be rather low [29]. Finally, we considered only binary problems, since these are the most common in the clinical context. Results might largely be different if multi-class problems are considered [59].

## 6. Future Work and Conclusions

Our study showed that the hope that deep features from pretrained networks are more predictive than hand-crafted features is unfounded. However, since we did not train and fine-tune the networks, their features were not specific to the dataset at hand; this could be a reason for the low predictive performance we observed. Therefore, training them could lead to models with higher predictive performance. This should be tested in a future study. Furthermore, we could not observe that networks pretrained on medical data outperformed those pretrained on ImageNet data in our study, which is unintuitive. The reasons for this are unclear, and a more thorough study on this should be performed, since a deeper understanding of this could be used to obtain more robust and predictive models. Our study did not analyze how far the features from different networks agree in terms of correlation. However, such an analysis could provide insights into why the fusion of hand-crafted and deep features did not yield a significant improvement. Finally, we did not test whether fusing features from different networks could be more helpful. This should also be tested in a future study.

In conclusion, our study showed that models based on features extracted from pretrained deep networks did not perform better on average. Fusing hand-crafted and deep features can yield some minor improvements, but will be specific to the dataset.

## Figures and Tables

**Table 1:** A list of studies between 2019 and 2022 that compare models based on hand-crafted features and deep features from pretrained networks.

Study	Year	Pathology	Modality	AUC (Hand-Crafted)	AUC (Deep)	ΔAUC
Zhu et al. [18]	2019	Brain cancer	MR	0.68	0.81	0.13
Feng et al. [19]	2020	Lung cancer	CT	0.7	0.8	0.1
Fu et al. [21]	2020	Rectal cancer	MR	0.64	0.73	0.09
Yan et al. [12]	2020	Cervical cancer	MR	0.72	0.72	0.0
Ziegelmayer et al. [20]	2020	Pancreatic cancer	CT	0.8	0.9	0.1
Aonpong et al. [13]	2021	Lung cancer	CT	0.68	0.69	0.01
Bo et al. [14]	2021	Brain cancer	MR	0.75	0.71	−0.04
Hou et al. [15]	2021	Prostate cancer	MR	0.83	0.84	0.01
Hu et al. [24]	2021	Lung cancer	CT	0.82	0.9	0.08
Xuan et al. [25]	2021	Placenta invasion	MR	0.8	0.88	0.08
Bertelli et al. [26]	2022	Prostate cancer	MR	0.8	0.85	0.05
Yang et al. [16]	2022	Head and neck cancer	CT	0.66	0.81	0.15
Yang et al. [17]	2022	Liver cancer	CT	0.74	0.88	0.14

ΔAUC denotes the difference in AUC between the model using hand-crafted and deep features.

**Table 2:** Datasets used for benchmarking.

Dataset	Modality (Weighting)	Sample Size (*n*)	Size of Minor Class	Size of Major Class	Class Balance	In-Plane Resolution [mm]	Slice Thickness [mm]	Source
C4KC-KiTS	CT	203	67	142	2.12	0.8 (0.4–1.0)	3.0 (1.0–5.0)	TCIA [31]
CRLM	CT	76	36	40	1.11	0.7 (0.6–0.9)	5.0 (1.0–8.0)	WORC [29]
Desmoid	MR (T1)	195	71	125	1.76	0.7 (0.2–1.8)	5.0 (1.0–10.0)	WORC [29]
GBM	MR (T1)	53	26	27	1.04	0.7 (0.6–0.9)	5.0 (1.0–8.0)	TCIA [32]
GIST	CT	244	120	125	1.04	0.8 (0.6–1.0)	3.0 (0.6–6.0)	WORC [29]
HN	CT	134	65	69	1.06	1.0 (1.0–1.1)	3.0 (1.5–3.0)	TCIA [33]
ISPY-1	MR (DCE)	157	69	92	1.33	0.8 (0.4–1.2)	2.1 (1.5–3.4)	TCIA [34]
Lipo	MR (T1)	113	57	57	1	0.7 (0.2–1.4)	5.5 (1.0–9.1)	WORC [29]
Liver	MR (T2)	186	92	94	1.02	0.8 (0.6–1.6)	7.7 (1.0–11.0)	WORC [29]
Melanoma	CT	97	48	49	1.02	0.8 (0.4–1.0)	3.0 (1.0–5.0)	WORC [29]

Class balance denotes the ratio of the major class to the minor class. DCE: Dynamic contrast enhanced; TCIA: The cancer imaging archive; WORC: Workflow for optimal radiomics classification.

**Table 3:** Performance metrics of the models based on hand-crafted and deep features, sorted by mean AUC.

Model	AUC	ΔAUC	Accuracy	ΔAccuracy	ΔRecall	Recall	Precision	ΔPrecision	MCC	ΔMCC	F1	ΔF1
Hand-Crafted Features												
Hand-crafted, 2-D, All	0.794	0.005	0.727	0.001	0.766	0.033	0.716	−0.013	0.441	0.002	0.74	0.01
Hand-crafted, 2-D, No morph	0.793	0.004	0.735	0.009	0.741	0.008	0.738	0.009	0.456	0.017	0.737	0.007
Hand-crafted, 3-D, No morph	0.791	0.002	0.733	0.007	0.732	−0.001	0.738	0.009	0.454	0.015	0.734	0.004
Hand-crafted, 3-D, All	0.789	-	0.726	-	0.733	-	0.729	-	0.439	-	0.73	-
Hand-crafted, 3-D, Morph	0.723	−0.066	0.663	−0.063	0.618	−0.115	0.698	−0.031	0.303	−0.136	0.627	−0.103
Deep Features												
ConvNeXt V2, large	0.775	−0.014	0.708	−0.018	0.718	−0.015	0.709	−0.02	0.402	−0.037	0.712	−0.018
SimSiam, ResNet-50	0.766	−0.023	0.704	−0.022	0.711	−0.022	0.704	−0.025	0.385	−0.054	0.699	−0.031
SimCLR, ResNet-50	0.761	−0.028	0.709	−0.017	0.745	0.012	0.701	−0.028	0.391	−0.048	0.72	−0.01
Deep and Morphological Features												
ConvNeXt V2, large	0.778	−0.011	0.714	−0.012	0.724	−0.009	0.717	−0.012	0.412	−0.027	0.72	−0.01
SimSiam, ResNet-50	0.777	−0.012	0.714	−0.012	0.745	0.012	0.707	−0.022	0.4	−0.039	0.723	−0.007
EfficientNet-B2	0.773	−0.016	0.716	−0.01	0.708	−0.025	0.73	0.001	0.416	−0.023	0.715	−0.015
Deep and All Hand-Crafted Features												
SimSiam, ResNet-50	0.798	0.009	0.734	0.008	0.727	−0.006	0.737	0.008	0.45	0.011	0.731	0.001
EfficientNet V2, large	0.795	0.006	0.719	−0.007	0.683	−0.05	0.669	−0.06	0.414	−0.025	0.674	−0.056
EfficientNet-B2	0.795	0.006	0.739	0.013	0.758	0.025	0.738	0.009	0.464	0.025	0.747	0.017

Models with “All” used all available hand-crafted features, while those with “No morph” used all but morphological features. The models with “Morph” used only morphological features. The “Hand-crafted, 3-D, All” model was considered to be the standard. All differences were computed relative to this model. Columns starting with Δ denote differences to the corresponding metric of the standard model, i.e., a value of 0.017 for ΔAccuracy means that the model performed 0.017 more accurately than the “Hand-crafted, 3-D, All” in mean over all datasets.

## Data Availability

Data and code can be found at https://github.com/aydindemircioglu/radSSL (accessed on 13 September 2023).

## Supplementary Materials

The following supporting information can be downloaded at: https://www.mdpi.com/article/10.3390/diagnostics13203266/s1, Table S1: List of pretrained deep networks used; Table S2: AUC of the models using hand-crafted features. Table S3. AUC of the models using deep features. Table S4. AUC of the models using deep features fused with morphological features. Table S5. AUC of the models using deep features fused with hand-crafted features were added.
