# Supplementary material for: Deep Features from Pretrained Networks Do Not Outperform Hand-Crafted Features in Radiomics
Source: Diagnostics (Basel). 2023 Oct 20;13(20):3266. doi: 10.3390/diagnostics13203266 (PMC10606594; doi:10.3390/diagnostics13203266)
Supplement: Supplementary file 1 [file diagnostics-13-03266-s001.zip › diagnostics-2638320-supplementary.pdf]

diagnostics

Deep Features from Pretrained Networks Do Not Outperform Hand-Crafted Features in Radiomics

Supplemental Material

Table S1. List of pretrained deep networks used.

Modelname	Pretraining data	Number of parameters	Size of feature vector
**radimagenet.resnet50**	Medical data	23,59	2048
**radimagenet.densenet121**	Medical data	7,04	1024
**radimagenet.inceptionV3**	Medical data	21,8	2048
**radimagenet.IRV2**	Medical data	54,33	1536
**medicalimagenet.resnet10**	Medical data	14,36	512
**medicalimagenet.resnet18**	Medical data	32,99	512
**medicalimagenet.resnet34**	Medical data	63,3	512
**medicalimagenet.resnet50**	Medical data	46,16	2048
**convnext-v2-large_fcmae-in21k-pre_3rdparty_in1k**	ImageNet-1K	197,96	1536
**deit3-huge-p14_in21k-pre_3rdparty_in1k**	ImageNet-1K	632,13	1280
**efficientnet-b7_3rdparty-ra-noisystudent_in1k**	ImageNet-1K	66,35	80
**efficientnetv2-l_in21k-pre_3rdparty_in1k**	ImageNet-1K	118,52	96
**simclr_resnet50_16xb256-coslr-200e_in1k**	Self-supervised	27,97	2048
**simsiam_resnet50_8xb32-coslr-200e_in1k**	Self-supervised	38,2	2048
**mocov3_resnet50_8xb512-amp-coslr-300e_in1k**	Self-supervised	68,01	2048
**barlowtwins_resnet50_8xb256-coslr-300e_in1k**	Self-supervised	174,54	2048
**resnet34_8xb32_in1k**	ImageNet-1K	2,18	512
**vgg16bn_8xb32_in1k**	ImageNet-1K	138,37	1000
**densenet161_3rdparty_in1k**	ImageNet-1K	28,68	2208
**efficientnet-b2_3rdparty_8xb32_in1k**	ImageNet-1K	9,11	48

Table S2. AUC of the models using hand-crafted features.

Model	C4KC-KiTS	CRLM	Desmoid	GBM	GIST	HN	I-SPY1	Lipo	Liver	Melanoma	Mean AUC	ΔAUC
**Hand-crafted, 2-D, All**	0.761	**0.79**	0.899	0.722	0.817	0.902	0.68	0.883	0.801	0.681	0.794	0.005
**Hand-crafted, 2-D, No morph**	0.756	0.778	0.891	0.741	**0.819**	0.902	0.68	**0.885**	**0.803**	0.681	0.793	0.005
**Hand-crafted, 3-D, No morph**	**0.768**	0.749	0.898	0.772	0.764	**0.914**	0.671	0.876	0.782	**0.719**	0.791	0.002
**Hand-crafted, 3-D, All**	**0.768**	0.749	**0.908**	**0.783**	0.78	0.903	0.671	0.883	0.774	0.669	0.789	0
**Hand-crafted, 3-D, Morph**	0.58	0.742	0.807	0.694	0.726	0.849	**0.681**	0.884	0.676	0.589	0.723	-0.066

Models with "All" used all available hand-crafted features, while those with "No morph" used all but morphological features. The models with "Morph" used only morphological features. ΔAUC denotes the mean difference to the standard "Hand-crafted, 3-D, All" model. Models with the highest AUC for each dataset were marked in bold.

Table S3. AUC of the models using deep features.

Model	C4KC-KiTS	CRLM	Des-moid	GBM	GIST	HN	I-SPY1	Lipo	Liver	Mela-noma	Mean AUC	ΔAUC
**ConvNeXt V2, large**	0.661	**0.821**	**0.881**	0.781	0.742	0.902	0.661	**0.886**	0.793	0.623	0.775	-0.014
**SimSiam, ResNet-50**	0.664	0.671	0.829	**0.915**	0.689	0.911	0.732	0.846	0.794	0.614	0.766	-0.022
**SimCLR, ResNet-50**	0.682	0.714	0.859	0.772	0.701	0.908	0.688	0.822	**0.819**	0.649	0.761	-0.027
**DenseNet-161**	0.675	0.676	0.867	0.709	**0.757**	0.89	0.688	0.843	0.778	0.666	0.755	-0.034
**ResNet-34**	**0.711**	0.743	0.845	0.645	0.738	0.874	0.652	0.885	0.816	0.607	0.752	-0.037
**VGG-16**	0.625	0.667	0.872	0.725	0.703	0.85	0.68	0.857	0.786	0.742	0.751	-0.038
**BarlowTwins, ResNet-50**	0.692	0.672	0.82	0.726	0.697	0.896	0.755	0.826	0.786	0.631	0.75	-0.039
**DeiT III huge**	0.646	0.689	0.761	0.785	0.738	0.864	**0.788**	0.871	0.702	0.644	0.749	-0.04
**EfficientNet-B2**	0.657	0.705	0.788	0.734	0.711	0.831	0.765	0.821	0.773	0.654	0.744	-0.045
**MoCo V3, ResNet-50**	0.67	0.734	0.872	0.651	0.685	**0.913**	0.674	0.827	0.779	0.6	0.74	-0.048
**EfficientNet-B7**	0.664	0.722	0.818	0.678	0.687	0.886	0.669	0.835	0.703	0.694	0.736	-0.053
**EfficientNet V2, large**	0.671	0.668	0.816	0.725	0.706	0.856	0.707	0.762	0.749	0.656	0.732	-0.057
**MedicalNet, ResNet-10**	0.688	0.652	0.749	0.819	0.736	0.812	0.645	0.758	0.726	0.712	0.73	-0.059
**MedicalNet, ResNet-50**	0.712	0.59	0.721	0.803	0.697	0.816	0.647	0.693	0.737	**0.775**	0.719	-0.07
**RadImageNet, InceptionRes-Net-V2**	0.61	0.669	0.743	0.756	0.689	0.744	0.663	0.743	0.645	**0.775**	0.704	-0.085
**RadImageNet, DenseNet-121**	0.61	0.684	0.746	0.699	0.706	0.751	0.707	0.778	0.719	0.599	0.7	-0.089
**MedicalNet, ResNet-18**	0.679	0.565	0.756	0.726	0.664	0.823	0.65	0.735	0.719	0.677	0.7	-0.089
**RadImageNet, Inception-V3**	0.599	0.642	0.774	0.781	0.669	0.78	0.638	0.75	0.688	0.576	0.69	-0.099
**MedicalNet, ResNet-34**	0.7	0.569	0.736	0.724	0.67	0.778	0.645	0.743	0.667	0.648	0.688	-0.101
**RadImageNet, ResNet-50**	0.642	0.715	0.691	0.766	0.676	0.714	0.688	0.702	0.598	0.662	0.685	-0.103

ΔAUC denotes the mean difference to the standard “Hand-crafted, 3-D, All” model. Models with the highest AUC for each dataset were marked in bold.

Table S4. AUC of the models using deep features fused with morphological features.

Model	C4KC-KiTS	CRLM	Desmoid	GBM	GIST	HN	I-SPY1	Lipo	Liver	Melanoma	Mean AUC	ΔAUC
**ConvNeXt V2, large**	0.661	**0.792**	**0.882**	0.801	0.746	0.902	0.674	0.884	**0.818**	0.618	0.778	-0.011
**SimSiam, ResNet-50**	0.699	0.671	0.851	**0.915**	0.675	0.902	0.732	0.884	0.793	0.644	0.777	-0.012
**EfficientNet-B2**	0.652	0.794	0.847	0.839	0.717	0.864	0.71	0.884	0.758	0.665	0.773	-0.016
**SimCLR, ResNet-50**	0.682	0.703	0.872	0.768	0.722	0.91	0.698	0.884	0.807	0.611	0.766	-0.023
**DenseNet-161**	0.675	0.684	0.867	0.731	**0.758**	0.896	0.688	0.884	0.778	0.67	0.763	-0.026
**BarlowTwins, ResNet-50**	0.692	0.68	0.855	0.726	0.707	0.904	0.744	0.884	0.782	0.628	0.76	-0.029
**VGG-16**	0.628	0.688	0.877	0.784	0.712	0.847	0.64	0.884	0.795	0.723	0.758	-0.031
**EfficientNet V2, large**	0.673	0.715	0.842	0.735	0.726	0.861	0.715	0.884	0.76	0.659	0.757	-0.032
**ResNet-34**	**0.715**	0.717	0.855	0.708	0.747	0.868	0.65	0.896	0.817	0.581	0.755	-0.033
**DeiT III huge**	0.646	0.71	0.773	0.798	0.741	0.865	**0.781**	**0.918**	0.678	0.644	0.755	-0.034
**MedicalNet, ResNet-50**	0.712	0.669	0.784	0.803	0.686	0.845	0.612	0.884	0.756	**0.776**	0.753	-0.036
**EfficientNet-B7**	0.664	0.742	0.842	0.714	0.725	0.875	0.663	0.884	0.713	0.695	0.752	-0.037
**MedicalNet, ResNet-10**	0.687	0.686	0.795	0.821	0.722	0.849	0.649	0.884	0.749	0.669	0.751	-0.038
**MoCo V3, ResNet-50**	0.67	0.72	0.876	0.651	0.699	**0.913**	0.681	0.884	0.779	0.618	0.749	-0.04
**RadImageNet, ResNet-50**	0.652	0.758	0.777	0.759	0.7	0.849	0.708	0.884	0.651	0.675	0.741	-0.048
**RadImageNet, InceptionRes-Net-V2**	0.666	0.66	0.781	0.752	0.706	0.841	0.673	0.888	0.654	0.775	0.74	-0.049
**MedicalNet, ResNet-18**	0.688	0.649	0.835	0.755	0.686	0.843	0.636	0.884	0.73	0.677	0.738	-0.051
**MedicalNet, ResNet-34**	0.7	0.633	0.818	0.738	0.697	0.849	0.644	0.884	0.727	0.648	0.734	-0.055
**RadImageNet, DenseNet-121**	0.602	0.685	0.77	0.689	0.748	0.836	0.664	0.887	0.709	0.606	0.72	-0.069
**RadImageNet, Inception-V3**	0.58	0.674	0.8	0.788	0.682	0.856	0.616	0.884	0.662	0.542	0.708	-0.08

ΔAUC denotes the mean difference to the standard "Hand-crafted, 3-D, All" model. Models with the highest AUC for each dataset were marked in bold.

Table S5. AUC of the models using deep features fused with hand-crafted features.

ModelName	C4KC-KiTS	CRLM	Des-moid	GBM	GIST	HN	I-SPY1	Lipo	Liver	Mela-noma	Mean AUC	ΔAUC
**SimSiam, ResNet-50**	0.74	0.711	0.868	**0.915**	0.768	0.904	**0.704**	**0.933**	0.794	0.647	0.798	0.009
**EfficientNet V2, large**	0.772	0.738	**0.925**	0.764	0.777	0.905	0.701	0.885	0.787	0.693	0.795	0.006
**EfficientNet-B2**	0.768	0.76	0.911	0.779	0.77	0.909	0.655	0.87	0.8	0.723	0.795	0.006
**MedicalNet, ResNet10**	0.754	0.749	0.891	0.819	0.78	0.896	0.649	0.873	0.779	0.73	0.792	0.003
**DeiT III huge**	0.759	0.753	0.852	0.754	**0.815**	0.882	0.701	0.884	0.788	0.718	0.791	0.002
**EfficientNet-B7**	0.771	0.772	0.923	0.754	0.762	0.906	0.676	0.873	0.768	0.699	0.79	0.001
**SimCLR, ResNet-50**	**0.794**	0.77	0.896	0.739	0.77	**0.911**	0.677	0.885	0.787	0.652	0.788	-0.001
**RadImageNet, ResNet50**	0.758	0.744	0.87	0.795	0.767	0.899	0.686	0.889	0.774	0.688	0.787	-0.002
**ConvNeXt V2, large**	0.743	**0.809**	0.911	0.751	0.781	0.902	0.656	0.882	0.789	0.643	0.787	-0.002
**MedicalNet, ResNet34**	0.771	0.767	0.883	0.781	0.782	0.899	0.671	0.885	0.766	0.657	0.786	-0.003
**ResNet-34**	0.763	0.74	0.895	0.765	0.791	0.889	0.643	0.886	**0.814**	0.644	0.783	-0.006
**BarlowTwins, ResNet-50**	0.737	0.749	0.889	0.749	0.785	0.886	0.701	0.875	0.785	0.674	0.783	-0.006
**VGG-16**	0.743	0.735	0.918	0.752	0.768	0.878	0.657	0.877	0.801	0.697	0.783	-0.006
**RadImageNet, InceptionRes-Net-V2**	0.758	0.742	0.876	0.701	0.761	0.899	0.683	0.873	0.774	**0.734**	0.78	-0.009
**MoCo V3, ResNet-50**	0.764	0.777	0.897	0.684	0.768	0.906	0.679	0.879	0.81	0.635	0.78	-0.009
**DenseNet-161**	0.78	0.735	0.909	0.697	0.795	0.89	0.669	0.884	0.803	0.631	0.779	-0.01
**RadImageNet, DenseNet-121**	0.745	0.784	0.866	0.749	0.784	0.899	0.664	0.874	0.764	0.662	0.779	-0.01
**MedicalNet, ResNet-18**	0.744	0.748	0.882	0.748	0.758	0.899	0.671	0.881	0.761	0.681	0.777	-0.012
**MedicalNet, ResNet-50**	0.739	0.725	0.866	0.747	0.775	0.897	0.619	0.876	0.795	0.712	0.775	-0.014
**RadImageNet, Inception-V3**	0.741	0.749	0.874	0.742	0.757	0.899	0.644	0.873	0.738	0.675	0.769	-0.02

ΔAUC denotes the mean difference to the standard "Hand-crafted, 3-D, All" model. Models with the highest AUC for each dataset were marked in bold.
